# Mechanisms and Advances in Anti-Ovarian Cancer with Natural Plants Component

**DOI:** 10.3390/molecules26195949

**Published:** 2021-09-30

**Authors:** Jingyuan Wu, Tuoyu Zhou, Yinxue Wang, Yanbiao Jiang, Yiqing Wang

**Affiliations:** 1 The First School of Clinical Medicine, Lanzhou University, Lanzhou 730000, China; Jywu16@lzu.edu.cn (J.W.); Jiangyb16@163.com (Y.J.); 2 Ministry of Education Key Laboratory of Cell Activities and Stress Adaptations, School of Life Sciences, Lanzhou University, Lanzhou 730000, China; zhouty19@lzu.edu.cn; 3 The Reproductive Medicine Special Hospital of the First Hospital of Lanzhou University, Lanzhou 730000, China; wangyx19@lzu.edu.cn; 4 Gansu Key Laboratory of Reproductive Medicine and Embryology, The First Hospital of Lanzhou University, Lanzhou 730000, China

**Keywords:** ovarian cancer, natural plant products, molecular mechanisms, bioactive compounds, traditional Chinese medicine

## Abstract

Ovarian cancer ranks seventh in the most common malignant tumors among female disease, which seriously threatens female reproductive health. It is characterized by hidden pathogenesis, missed diagnosis, high reoccurrence rate, and poor prognosis. In clinic, the first-line treatment prioritized debulking surgery with paclitaxel-based chemotherapy. The harsh truth is that female patients are prone to relapse due to the dissemination of tumor cells and drug resistance. In these circumstances, the development of new therapy strategies combined with traditional approaches is conductive to improving the quality of treatment. Among numerous drug resources, botanical compounds have unique advantages due to their potentials in multitarget functions, long application history, and wide availability. Previous studies have revealed the therapeutic effects of bioactive plant components in ovarian cancer. These natural ingredients act as part of the initial treatment or an auxiliary option for maintenance therapy, further reducing the tumor and metastatic burden. In this review, we summarized the functions and mechanisms of natural botanical components applied in human ovarian cancer. We focused on the molecular mechanisms of cell apoptosis, autophagy, RNA and DNA lesion, ROS damage, and the multiple-drug resistance. We aim to provide a theoretical reference for in-depth drug research so as to manage ovarian cancer better in clinic.

## 1. Cognitive Status of Ovarian Cancer

Ovarian cancer is the seventh most common malignant tumor cancer and the fifth leading cause of death among female reproductive diseases [1]. The number of cases diagnosed is steadily increasing with the higher life expectancy, while wildly recognized preventative measures or effective treatments have not kept up yet. The disease is not specifically diagnosed until an advanced stage, causing a relatively high morbidity and mortality. Correspondingly, symptoms progress insidiously as tumorigenesis, including pelvis pain and lower abdomen pain, vaginal bleeding during menstrual periods, unregular bleeding after menopause, bloating, and changes in urinary or bowel habits. In some cases, the primary cancer cells attack adjacent tissue and spread to distant organs. The metastasis of ovarian cancerous cells in the peritoneal cavity actually move for a long time without obvious symptoms, and the routes of dissemination of ovarian cancer include intracavitary implantation, hematogenous metastasis, and lymphatic metastasis [2]. On the one hand, its threatening aggressiveness relies on the rapid dissemination of cancer cells to adjacent tissues, such as the peritoneum, the omentum, and abdominal organs. On the other hand, the development of drug resistance to chemotherapeutic agents cannot be neglected. Histopathologically, nearly 90% of ovarian cancer originates from the ovarian surface epithelium (OSE), with the rest being classified as serous, mucinous, endometrioid, transitional, and clear cell carcinomas [3,4]. It is a heterogeneous disease involved with inherited mutations in susceptibility genes, such as p53 tumor suppressor gene, ERBB2, and PIK3CA oncogenes, etc. Approximately 10% of ovarian cancer occurs in women carrying BRCA1 or BRCA2 mutations [5,6], causing some ovarian cancers clustering in families. Moreover, various classic signaling pathways participate in oncogenesis. For instance, Wnt/β-catenin target genes mediate cancer initiation and progression. PI3K/AKT/mTOR plays a regulatory role in cell survival, growth, and proliferation [7,8]. Notch signal pathway is associated with cell proliferation, migration, cell stemness, and chemoresistance [9]. Hyperactivation of Hedgehog signal transduction also participates in chemotherapy-resistant phenotype, because the overexpression of transcription factor is connected to the regulation of ABCB1 and ABCG2 gene expression in ovarian cancer [10].

## 2. Current Treatments for Ovarian Cancer

At present, clinic treatment prioritized debulking surgery along with platinum-based chemotherapy, for example, cyclophosphamide and doxorubicin hydrochloride. The standard chemotherapy regimens are taxanes including paclitaxel and docetaxel and platinum agents such as carboplatin and cisplatin [11]. In combination with paclitaxel and carboplatin, humanized monoclonal antibody bevacizumab is administered for patients with advanced ovarian cancers. The harsh truth is that quite a number of patients who received and responded to chemotherapy are prone to develop chemotherapy resistance. Moreover, the cumulative toxic effects included but were not limited to nephrotoxicity, neurotoxicity, ototoxicity with cisplatin, and myelosuppression with carboplatin [12]. Although early treatment is effective for most patients, relapse is prone to occur within a median of 16 months in advanced-stage patients [13]. In particular, the genetic alterations lead to increasing repairments of DNA lesions, derangements of intracellular signal transduction, and drug metabolic inactivation. The success of complete remission relied on surgical skills accompanied with the gene signature of the tumor [14]. Patients who carried BRCA1 or BRCA2 germline mutations are recommended for olaparib administration as a poly ADP-ribose polymerase (PARP) inhibitor [15]. It is also applied for relapsed and subsequent platinum-based chemotherapy.

However, once it relapses, the interval between subsequent treatments declines steadily due to rapid tumor progression and chemotherapy resistance. Novel options rather than only traditional chemotherapy are needed to improve the survival quality of patients for systemic treatment. Among the potential drug resources, botanical components are natural antioxidant constituents due to their long history in ethnopharmacology. Conventionally, plant-derived natural products are considered to be auxiliary nutritional supplementary. Meanwhile, as part of an initial treatment or an option for maintenance therapy, the effective components from natural plants could improve multiple-drug sensitivity, further reducing the tumor and metastatic burden of ovarian cancer. In traditional Chinese medicine, triptolide is a herb-derived antineoplastic agent, which has been proved to selectively kill both p53 mutated and p53 wild-type ovarian cancerous cells [16]. Emodin is a natural anthraquinone extracted from traditional Chinese herbs, which was identified to inhibit the growth of human ovarian carcinomas [17]. Potential herbal remedies have advantages in wide availability, few side effects, and are generally well tolerated. From experimental findings to clinical applications, classic herbal extracts have been broadly used for management in ovarian cancer.

## 3. Mechanisms with Natural Plants Compound in Ovarian Cancer Cells

### 3.1. Cytotoxic Effect

Cryptotanshinone, tanshinone-I, and tanshinone-IIA are natural components mainly isolated from the roots of Salvia miltiorrhiza Bunge, which is named Danshen in traditional Chinese herbs [18]. TNF-related apoptosis-inducing ligand (TRAIL) is known as ‘death receptors’ in the TNF superfamily, which initiates the cell death progression by binding to its associated receptor [19,20]. In TRAIL-resistant cells A549 and human ovarian cancer cells TOV-21G, exposure with three tanshinones effectively enhanced the functionality of TRAIL, thus reducing cell viability and inhibiting the colony formation capacity. Especially, tanshinone-IIA had the most potential among all for displaying an IC_50_ of 2.00 ± 0.36 μM and 2.75 ± 0.23 μM in both A549 and TOV-21G cell lines [21]. In a dose-dependent pattern, TII-A enhanced JNK-mediated signaling transduction. TII-A augmented the expression levels of DR5 and mRNA, leading to TRAIL-sensitive cytotoxicity [22]. Kadsuphilactone B is a nortriterpenoid compound derived from traditional Chinese herb Schisandra chinensis (Turcz) Baill. In ovarian cancer cells A2780 and Ishikawa, it exhibited a cytotoxic effect with IC50 values below 25 μM. In a dose-dependent pattern, kadsuphilactone B promoted the stimulations of caspase-3/8/9, MAPKs, cleaved PARP, and alterations in Bcl-2 family proteins levels [23]. Methyl lucidone (ML) is an herbal component contained in the dried fruits of *L. erythrocarpa* Makino. In SKOV-3 and OVCAR-8 cell lines, ML exhibited cytotoxic effects by displaying the IC_50_ of 48.8–60.7 and 33.3–54.7µM. Treatment with ML induced obvious cellular apoptosis and morphological changes. ML not only stimulated the activation of cleaved caspase-3/9 and PARP but also released cytochrome c in mitochondria. In addition, ML diminished the protein expression levels of Bcl-2 family, including Bcl-2 and Bcl-xL [24].

### 3.2. Inhibit Proliferation and Promote Apoptosis

Apoptosis is a conserved form of programmed cell death required for homeostasis, whereas in noncontrolled situations could be a major cause for tumorigenesis [25]. Zeylenone is a naturally occurring cyclohexene oxide extracted from the leaves of Uvaria grandiflora Roxb. of the family Annonaceae [26,27]. In SKOV3 cells incubated with zeylenone at different concentrations (2.5, 5, and 10 μmol/L for 24 h), Janus family of tyrosine kinase (p-JAK), and signal transducer with activator of transcription (p-STAT) expression levels were reduced variously. In a dose-dependent pattern, zeylenone increased the loss of MMP, apoptosis-inducing factor (AIF), and cytochrome c. Apart from that, it increased caspase-3, Fas, Fasl, and Bax in both the mRNA and protein expression levels but decreased Bcl-2 expression level [28].

Berbamine is a plant-derived component isolated from the traditional Chinese medicine *Berberis amurensis*, which is already applied for leukemia treatment in clinic. In ovarian cancer cells SKOV-3, berbamine upregulated the cleaved caspase-3, cleaved caspase-9, and Bax in protein expression levels, while it downregulated the antiapoptotic protein level of Bcl-2 family. Berbamine inhibited cell proliferation and promoted apoptosis by partially suppressing the Wnt/β-catenin signaling transduction [29]. *Pinus massoniana* bark proanthocyanidins (PMBP) is a bioactive compound derived from natural root bark of Pinus massoniana. In A-2780 and OV2008, PMBP downregulated the expression level of Bcl-2 family and increased the levels of Caspase-3/9. PMBP significantly repressed MMP-9, ERK1/2, and p38 MAPK expression, thus blocking the activity of NF-κB. Furthermore, it triggered activations of mitochondria-associated apoptosis through the loss of mitochondrial membrane potential [30]. Sanguiin H-6 is a natural component derived from red raspberry fruits. In A2780 cells incubated with Sanguiin H-6 at concentrations of 20/40 μM, the early apoptotic percentages differently exhibited at 35.39 and 41.76. Sanguiin H-6 triggered the stimulation of MAPK p38 through a caspase-8-dependent BID cleavage signaling pathway. Furthermore, Sanguiin H-6 promoted the antiproliferative effect by activations of caspases. It is also observed a severe morphological change and more cleaved PARP, which is known as the biomarker of apoptosis [31].

### 3.3. Suppress Cell Migration and Invasion

The epithelial cells transdifferentiated into motile mesenchymal cells, which is known as epithelial-mesenchymal transition (EMT) [32]. EMT is a self-regulated process in physiological conditions, whereas EMT might step to cancer in pathological situations [33]. Tetramethylpyrazine (TMP) is a widely used medicinal compound extracted from Ligusticum Wallichil Franch. In SKOV-3 and OVCAR-3 cells, TMP significantly increased the expression level of miR-211, which could be partially abrogated by the knockdown of miR-211 [34]. TMP inhibited the cell viability, migration, and invasion ability of both cell lines in a dose-dependent pattern. Treatment with TMP at various concentrations (25–100 µg/mL) in SKOV-3 for 24 h exhibited the downregulation of IL-8 accompanied with the suppression of cell migration [35].

Dihydroartemisinin (DHA) extracted from the traditional Chinese herb Artemisia annua is widely used for malaria treatment. Unusual activation of the Hedgehog (Hh) signaling transduction is generally associated with human ovarian cancer tumorigenesis. In ovarian cancer cells, DHA obviously inhibited cell malignant behaviors and also augmented cell apoptosis. By contrast, DHA had few effects in human ovarian surface epithelial cells (HOSEPICs) in vitro. Specifically, DHA suppressed the Hh signaling pathway by inhibiting its agonist purmorphamine; in addition, DHA enhanced the Hh signaling pathway inhibitor GANT61 [36]. Emodin is a natural anthraquinone contained in various Chinese medicine herbal preparation herbs, for instance, *Rheum palmatum*, *Polygonum cuspidatum*, and *Polygonum multiflorum* [37]. In A2780 and SKOV-3 cells, it has been proven that emodin specially suppressed the abilities including cell proliferation, invasion, and migration. In a dose-dependent pattern, emodin treatment led to upregulation of epithelial markers, such as E-cadherin and Claudin. It also resulted in downregulation of mesenchymal markers, such as N-cadherin, Vimentin, and transcription factor Slug. Moreover, emodin diminished ILK, p-GSK-3β, β-catenin, and Slug expression levels, producing similar effects of siRNA transfection. These results could be exemplified by the following emodin incubation. It revealed that emodin repressed the EMT of ovarian cancer cells through ILK/GSK-3β/Slug signaling transduction [38].

### 3.4. Reactive Oxygen Species (ROS) Damage

From inflammation to cancer, excessive oxidative stress is deemed as a regulatory factor in multiple diseases. Although partial signaling transductions still need to be explored, clear molecular mechanisms for diseases progression have been elucidated. The association from excessive reactive oxygen species (ROS) damage to tumorigenesis implied the importance of antioxidant therapy [39]. Sulforaphane (SFN) is a phytochemical component contained in cruciferous plants such as broccoli. In cell lines OVCAR3, OVCAR4, OVCAR5, and SKOV3, SFN significantly promoted apoptosis at various concentrations. On the other hand, xenograft experiments in vivo elucidated that SFN effectively suppressed tumor growth through targeting tumor-related signaling transduction, while SFN did not produce consequential levels of ROS in cells. Moreover, a thiol-oxidizing agent named diamide enhanced the effect present by SFN, indicating protein thiols oxidation and/or cellular redox status changes may be an underlying mechanism [40].

Quercetin is a prominent dietary flavonoid, which ubiquitously presents as an antioxidant in onion, milk thistle, and French maritime pine bark [41]. In SKOV3 and OVCAR3 cell lines, quercetin increased apoptosis through the stimulation of transcription factor CCAAT enhancer-binding protein homologous protein (CHOP). The expression level of death receptor DR5 was induced by CHOP, followed with ROS-mediated endoplasmic reticulum-stress damage. In the human SKOV-3 xenograft model, quercetin inhibited tumor growth mediated by TRAIL sensitization. It was synergistically accompanied by the induction of apoptosis and the activation of caspase-3 [42]. Formononetin (FMN) is an isoflavone contained in red clovers and soy, which exhibits antioxidant and anticancer capacities in various cell types. In ES2 and OV90 cell lines, FMN augmented the loss of mitochondrial membrane potential as well as generation ROS. Additionally, FMN suppressed cell proliferation and induced apoptosis via interfering with sub G0/G1 cell cycle phase arrest, accompanied by decreasing phosphorylation levels of ERK1/2, P90RSK, P70S6K, and AKT and increasing the phosphorylation level of P38 protein. In both cell types, incubation of FMN simultaneous with pharmacological inhibitors LY294002/U0126 resulted in additional antiproliferative outcomes. [43].

### 3.5. Trigger Cell Cycle Arrest

Although ovarian cancer possesses its complexity and idiopathy, one contributing factor propelled the tumorigenesis is deregulated cell cycles [44]. To unravel the dynamics of cell cycle arrest, dysregulation presents a better understanding in human cancer cell lines. Cucurbitacin-A, isolated from *Momordica charantia* L. (M. charantia), has been identified with anticancer and anti-inflammatory activities [45]. In SKOV3 cell line, cucurbitacin-A exhibited an IC50 of 40 μM and triggered the G2/M checkpoint in cell cycle arrest. It caused DNA lesions and prompted mitochondrial membrane potential loss (MMP) through ROS-mediated alterations. Moreover, it considerably suppressed the expression levels of several proteins of PI3K/Akt/mTOR signaling transduction [46]. Asiatic acid is a natural triterpenoid component enriched in the tropical medicinal herb Centella asiatica. In SKOV3 and OVCAR-3 cell lines, incubation with Asiatic acid (10–100 μg/mL for 48/72 h) significantly downregulated the phosphorylation levels of PI3K, AKT, and mTOR. Both cell lines treated with Asiatic acid exhibited an increasing 7–10-fold apoptosis at G0/G1 phase cell cycle arrest. The colony formation of cells was remarkably reduced by 25–30% at a specific concentration of 10 μg/mL. Satisfyingly, the ovarian cancer cells viability was reduced by 50%, and little effect was observed on human ovarian epithelial cells [47]. 

Amentoflavone (AF) is one of the natural herbal bioflavonoids extracted from Selaginella tamariscina [48]. In SKOV3 and OVCAR-3 cell lines, AF downregulated the expression levels of S-phase kinase protein 2 (Skp2), in which progression was regulated through ROS/AMPK/mTOR signaling pathway. In a dose-dependent pattern, AF incubation for 48 h triggered G1/G0 phase cell cycle arrest. In a xenograft animal model, AF could also significantly induced apoptosis in vivo [49]. Proanthocyanidins (BLPs) is isolated from Chinese bayberry leaves (Myrica rubra Sieb. et Zucc.). In chemotherapy-resistant OVCAR-3 spheroid cells, BLPs could attenuate chemotherapy-resistant traits. BLPs showed repressive effects on cell growth and cancer stemness characteristics. BLPs inhibited the abilities of cell viability, sphere and colony formation, and expression of stemness-related proteins. Specifically, BLPs targeted the Wnt/β-catenin signaling pathway by decreasing the expression of β-catenin, cyclin D1, and c-Myc and induced G1 phase cell cycle arrest, further inhibiting the capacity of self-renewal [50]. The traditional Chinese herbs Scutellaria baicalensis (SB) exhibited significant activities in treating hepatitis and diarrhea. Fritillaria cirrhosa (FC) is also broadly used in respiratory diseases for a long history. In ovarian cancer cells OVCA 420 and endometrial cancer cell OVCA 429, cotreatment with either herb interfered with G0/G1 phase cell cycle arrest. Both of SB- and FC-induced p27 expression, caused the activation of caspase-3, along with downregulation of cyclin D1 and cyclin D3. Furthermore, both herbs abrogated the activation of NF-κB and downregulated metastasis-promoting proteins [51].

### 3.6. Induce Autophagic Cell Death

Autophagy, unlike the apoptosis mentioned above, is relatively a conserved pathway. Materials in cytoplasm are transmitted to lysosomes for degradation by targeted enzymes. It is activated by quite a lot of stimuli, including hypoxia, protein aggregates, and oxidative stress in response to drugs and radiation [52]. Tanshinone I (Tan-I), an essential fat-soluble monomer component, is derived from Chinese folk herbs DanShen. In cell lines A2780 and ID-8, incubation with Tan-Ⅰ (0, 1.2, 2.4, 4.8, and 9.6 μg/mL for 24 h) augmented the protein levels of apoptosis-associated protein Caspase-3 and autophagic-associated proteins, for instance Beclin1, ATG7, p62, and LC3II/LC3I. The PI3K/AKT/mTOR signaling transduction were also upregulated simultaneously. The autophagic vacuoles were observed, which demonstrated that Tan-Ⅰ promoted apoptosis, activated the cleavage of caspase-3, and induced autophagy in human ovarian cancer [53]. Grifolin is one of the secondary metabolic byproducts contained in the polypore mushroom Albatrellus, which had potential in treating nasopharyngeal carcinoma. In both cell lines A2780 and SKOV3, grifolin induced autophagic cell death and inhibited cell proliferation. Grifolin treatment also upregulated expression levels of autophagy markers, for instance LC3B, Atg7, and Beclin-1. It downregulated the expression level of P62 and suppressed the proteins of p-Akt, p-mTOR, p-p70S6K, and p-4E-BP1. In addition, grifolin regulated the AKT/mTOR/S6K signaling transduction in autophagy of human ovarian cancer [54].

Genistein is a phenolic compound abundant in soy-based products, which possess the characteristics of antitumor in various cancer types [55]. In cisplatin sensitive and resistant (A2780/CaOV3, ES-2) ovarian cancer cells, different concentrations of genistein treatment (0.1–10 μM for 24–96 h) led to both cell apoptosis and autophagy. Genistein caused caspase-independent cell autophagy in a dose-dependent manner. Genistein also regulated substrate for glucose uptake, oxidative phosphorylation, and fatty acid synthesis. In addition, genistein downregulated the expression level of phosphorylated Akt, which involved in mechanisms of limited glucose utilization [56]. Isoliquiritigenin (ISL) is a flavonoid component enriched in the licorice plants. In OVCAR-5 and ES-2 cell lines, ISL significantly suppressed the cell viability in a both concentration- and time-dependent pattern. ISL promoted the expression levels of apoptosis and autophagy proteins, including cleaved PARP, cleaved caspase-3, Bax/Bcl-2 ratio, LC3B-II, and Beclin-1. Moreover, ISL triggered the G2/M phase cell cycle arrest [57].

### 3.7. Inhibit Angiogenesis

Chinese bayberry leaves proanthocyanidin (BLP) is a major effective compound isolated from Chinese bayberry. In ovarian cell line IOSE-364, BLP exhibited inhibitory effects of angiogenesis. It triggered G1 phase cell cycle arrest by repressing the protein levels of c-Myc, cyclin D1, and CDK4. In cisplatin resistant A2780 and CP70 cells, BLP especially attenuated the ability of wound healing. Furthermore, BLP reduced the expression level of HIF-1α. It decreased the level of angiogenesis-associated biomarker vascular endothelial growth factor (VEGF). BLP also suppressed ROS generation and regulated Akt/mTOR signaling transduction in human ovarian cancer [58]. Six natural bioactive flavonoids, including apigenin, taxifolin, luteolin, quercetin, genistein, and kaempferol, are all water-soluble vitamins. In OVCAR-3 cell line, six flavonoids suppressed the expression of VEGF in a dose-dependent manner. In addition, six plant-derived flavonoids inhibited the ovarian cancer cell proliferation [59].

Baicalin and its aglycone baicalein are specially enriched in traditional Chinese herbs Scutellaria [60]. In OVCAR-3 and CP-70 cell lines, either baicalin or baicalein reduced cell viability with LD₅₀ at a median of 50 and 32.5 µM. In normal ovarian cell line IOSE-364, both natural components exerted less inhibitory effects. Baicalin and baicalein suppressed cancer promoting genes such as VEGF, HIF-1α, c-Myc, and NF-kB at different concentrations, thus inhibiting the cell viability. Specifically, baicalein achieved the same effect at a lower concentration in both ovarian cancer cell lines [61]. Harmine (HM) is a small-molecular β-carbolinujhe alkaloid present in medicinal plants, which has been applied in folk medicine for antitumor therapy. In SKOV-3 cell line, HM remarkably downregulated the cell proliferation in a dose-dependent pattern. It also suppressed the cell proliferation and migration regulated by epidermal growth factor (EGF). Furthermore, HM inhibited the concentrations of ERK1/2 and CREB in both the basal level and EGF-induced phosphorylation level. HM significantly downregulated the expression level of VEGF and MMP-2/9 [62]. Cranberry proanthocyanidin-1(PAC-1) is abundant in cranberry, which targets ovarian cancer viability due to its cytotoxic and angiogenesis properties. In chemotherapy-resistant SKOV-3 cell lines, PAC-1 blocked G2/M phase cell cycle progression and increased ROS generation in a dose-dependent pattern. It induced apoptosis in both intrinsic and extrinsic pathway. In human umbilical vein endothelial cell line (HUVEC), PAC-1 suppressed the cell viability significantly. Moreover, PAC-1 interfered with the phosphorylation level of VEGF-stimulated receptor, thus inhibiting the formation of endothelial tube [63]. 

### 3.8. Interfere with RNA Expression

The discovery of aberrant RNAs play important oncogenic roles in various human cancers. These RNAs and their connections with gene expressions may provide the new sight to the detection, diagnosis, and targeted therapeutics for human ovarian cancer. Astragalus polysaccharide (APS) is a bioactive component isolated from a major traditional herb Astragalus membranaceus, which is named HuangQi in Chinese folk medicine. In OV-90 and SKOV-3 cell lines, APS repressed the cell proliferation and induced apoptosis. F-box and WD-40 domain protein 7 (FBXW7) is a star tumor inhibitor, which translation process is targeted and suppressed by miR-27a directly. In both cell lines, miR-27a upregulation obviously reversed the inhibitory effects exhibited by APS, such as antiproliferation and proapoptotic. It produced the same as the effects of knockdown of FBXW7 by si-FBXW7 [64]. Tanshinone IIA (Tan-IIA) is a natural plant-derived component enriched in Chinese herbal medicine DanShen. In the A2780 xenograft model, Tan-IIA suppressed tumor growth significantly. In ID-8 and A2780 cell lines, Tan-IIA induced apoptosis and promoted antiangiogenesis in a dose-dependent pattern. Tan-IIA also triggered G2/M phase cell cycle arrest. It downregulated expression level of Bcl-2 and upregulated level of Bax. Tan-IIA also decreased VEGF and cyclooxygenase-2 (COX2) mRNA expressions to promote antiangiogenesis [65].

### 3.9. Promote DNA Damage Response

**Figure 1:**
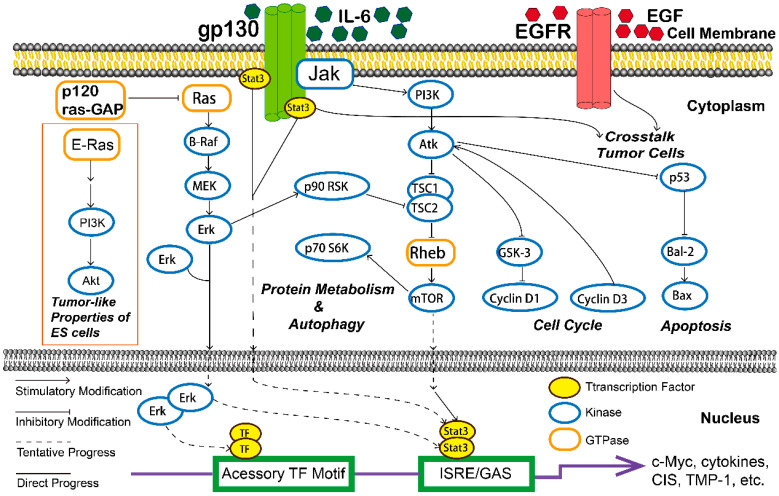
The various cellular signaling pathways of natural plants components in ovarian cancer.

Platinum-based drugs, radiation, and many other chemotherapeutic agents could induce DNA damages directly or indirectly. The ability of ovarian cancer cells to repair DNA lesions partly determined the efficiency of chemotherapeutic drugs [66]. Sideroxylin is a natural bioactive flavone contained in Callistemon lanceolatus leaves, which exhibits anti-inflammation activity against the pathogen *S. aureus*. In ES2 and OV90 cell lines, sideroxylin significantly suppressed cell proliferation and induced apoptosis. Sideroxylin at different concentrations stimulated the phosphorylation levels of proteins including ERK1/2, JNK, P38, and MAPK. Furthermore, it caused DNA fragmentation and depolarization of mitochondrial membrane. It increased more lipid peroxidation and the generation of ROS through PI3K/MAPK signal transduction [67]. Epigallocatechin gallate (EGCG) is one of the major components in green tea. Sulforaphane (SFN) is extracted from cruciferous vegetables. In paclitaxel-sensitive and -resistant ovarian cancer cell lines (SKOV3TR-ip1/SKOV3-ip2), SFN suppressed cell viability in a time- and dose-dependent pattern. Furthermore, EGCG potentiates the outcomes triggered by SFN. The cotreatment resulted in both G2/M along with S phase cell cycle arrest and decreased Bcl-2 protein levels. Treatment combined with both components in SKOV3TR-cells increased apoptosis and reduced hTERT concentration, which acted as major regulatory subunit in telomerase. Therefore, EGCG and SFN treatment promoted DNA damage response especially in paclitaxel-resistant ovarian cancer cell lines [68]. Berberine is an alkaloid component extracted from various herbal plants, which was proved to inhibit cell proliferation. In three cell lines A2780, HEY, and HO8910, berberine triggered DNA damages and repressed homologous recombination repair (HRR). PARP1 is a protein that senses the oxidative DNA-strand damages and coordinates their restorations. Correspondingly, berberine hyperactivated PARP1 in the A2780 as well as HO8910 cell lines. In coincubation of berberine and PARP inhibitor niraparib, tumor progression was remarkably diminished in vivo. Thus, berberine sensitized ovarian cancer cells to PARP inhibition by causing oxidative DNA damages and repressing HRR [69]. The detail effects and various cellular signaling pathways were summarized in Table 1 and Figure 1.

## 4. Mechanisms of Botanical Components Improve Drug Sensitivity

### 4.1. Modulate Immune Cell Responses

Considerable progress in elucidating how cancers escape from destructive immunity has been made, yet measures to solve tumor evade have not kept up [77]. As previous studies have illustrated, the activations of natural killer T (NKT) cells are inhibited immune responses and antitumor activities. As mentioned above, genistein induced ovarian cancer cells in autophagic. In OVCAR-3 and SKOV-3 cell lines, treatment with bevacizumab and genistein caused downregulation in ganglioside GD3 levels and repair of NKT-cell functions. Coincubation with bevacizumab and genistein also suppressed the VEGF, which was associated with ovarian cancer cells [71]. Natural herbal carotenoids, such as β-carotene, lutein, zeaxanthin, and curcumin, contained in retinoids-rich vegetables and fruits. In both a time- and dose-dependent pattern, carotenoids stimulated the activities of immune cells including lymphocytes, macrophages, and cytotoxic T-cells. Epidemiological statistics proved that supplementation with high carotenoid intake concomitants with a decreased risk of ovarian cancers [72].

### 4.2. Regulate Immune Molecular Expression Level

It has been shown that immune cells would alter functional phenotypes when cancer cells signal diversely. The immune molecules are a key factor in the tumor microenvironment. Lycopene, a bioactive natural compound in tomato, possessed the highest antioxidant properties among carotenoids [78]. In both ovarian cancer-bearing mice and established ovarian cancer cell models, lycopene effectively diminished the tumor load. In OV-MZ-6 cell lines, lycopene decreased expression levels of ITGA5, ITGB1, MMP9, FAK, ILK, and EMT biomarkers. It downregulated protein expressions of integrin α5 and activations of MAPK. Moreover, lycopene enhanced antitumorigenic concomitantly with antimetastatic effects of paclitaxel and carboplatin [73]. Deoxyschizandrin is an active lignin ingredient abundant in Schisandra Chinensis (Turcz.) Baill and Schisandra berries have been proven to possess antioxidant effect in previous studies [79]. In A-2780 cell lines, deoxyschizandrin arrested the G_0_/G_1_ phase cell cycle and interfered with the cyclin E expression level. It increased the production of ROS and decreased the activation of Akt. It also inhibited macrophages, which were activated by the ovarian cancer cells. Moreover, deoxyschizandrin inhibited the expression level of the M2 phenotype markers CD163 and CD209 in tumor-associated macrophages (TAMs). The tumor-promoting factors MMP-9, RANTES, and VEGF were significantly enhanced in TAMs, while the expression and production levels of these immune molecules were all suppressed by deoxyschizandrin effectively [74].

### 4.3. Reverse Multiple-Drug Resistance (MDR)

**Figure 2:**
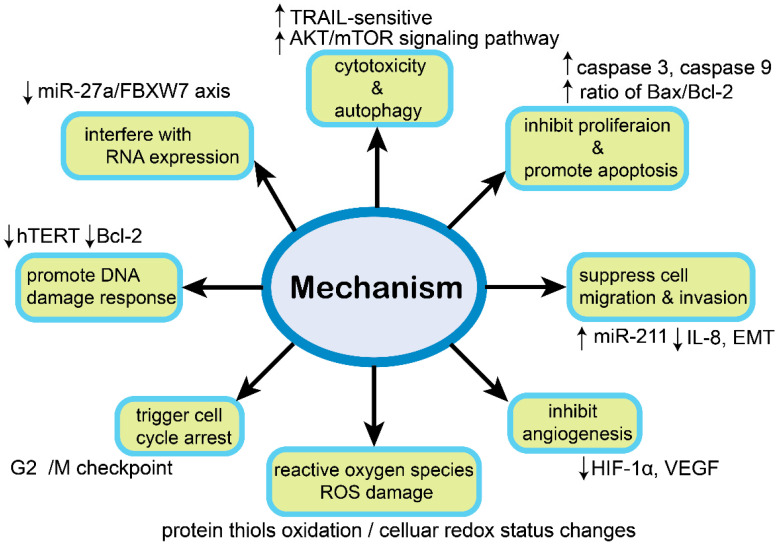
The role of molecular mechanisms in ovarian cancer.

Drug resistance is a troublesome phenomenon when proceeding the treatment of classical platinum drugs in ovarian cancer. Accordingly, there are two mechanisms of chemoresistance categories classified by most studies, including de novo (intrinsic) and acquired (extrinsic). However, the detailed reasons have not been illustrated comprehensively [80]. Curcumin is often named ‘curry powder’ in daily life, which is popularly in Indian spice for cooking. It is mainly derived from plant curcuma longa and well known as turmeric in constituent folk medicine [81]. In two in vivo models of SKOV3ip1 and HeyA8, curcumin led to a 49% and 55% reduction in average tumor growth compared with controls. When coincubated along with docetaxel, the number ranked at 96% and 77% significantly. In multidrug-resistant cell line HeyA8-MDR, curcumin alone and in combination with docetaxel exhibited 47% and 58% average reductions in tumor growth. In addition, both of the treatments reduced cell proliferation, microvessel density, and increased tumor cell apoptosis [75]. Tetramethylpyrazine (TMP), enriched in natural herb Ligusticum hort, was identified to act as a multilink role in the anticancer process. In A-2780 and SKOV-3 cell lines, the antitumor effects of paclitaxel were augmented by TMP. Treatment of TMP and paclitaxel inhibited signal transduction of ERK1/2 and Akt, promoted cancer cell apoptosis, and suppressed angiogenesis. In A-2780 xenograft mouse models, TMP potentiated the decrease in the tumor burden induced by paclitaxel. Moreover, TMP partially decreased the drug toxicity of paclitaxel [70]. Procyanidin (GSP) is a natural effective polyphenol compound abundant in grape seed [79]. In both A-2780 and paclitaxel-resistant A-2780/T cell lines, GSP significantly augmented the cytotoxicity of paclitaxel and adriamycin. P-glycoprotein (P-gp) is a protein related to the phenotype of MDR. GSP reversed ovarian cancer MDR by suppressing the transcription and expression of P-gp. Moreover, it downregulated the activation of NF-κB and MAPK/ERK pathway [76]. The molecular mechanisms are shown in Figure 2. 

## 5. Antitumor Effects and Chemical Structure Relationship in Natural Plants Compounds 

In treating ovarian cancer, although some natural bioactive components have already been applied clinically, the widespread adoption of them is not quite optimistic for several parameters. It is dragged down by weak solubility, uncontrolled stability, and poor bioavailability. The nonspecific distribution, short retention time, and rapid rate of elimination are also limiting factors [82]. Accordingly, investigating the structure generalities and specialties of effective components might be the breakthrough in ovarian cancer treatment. Based on the preliminary analysis of natural bioactive compounds in antiovarian cancer, we summarized several potential target structures including flavonoids, phenols, alkaloids, terpenoids, quinones, and esters. Kadsuphilactone B, cucurbitacin-A, grifolin, and lycopene are classified as terpenoids, which are familiar in daily diet tastes. Terpenoids possess the same precursor as phytocannabinoids, which is a star agent for cancer pain alleviation [83]. The synergistic relationships of the terpenoids and other drugs used in ovarian cancer indicated that some natural bioactive components might also retain unknown effective properties in human body, for the shared structure determine analogous functions. The chemical structures of effective compounds hinted that most of them contain the exact or potential polyphenol structure and alkaloids containing nitrogen. The structural formula of formononetin, genistein, emodin, proanthocyanidins, and other flavonoids carry with arrangements of polyphenol structure in meta-, ortho-, and para-position. In addition, curcumin contains a benzene ring connected with methylol groups, which probably lead to hydrolytic reactions and phenolic hydroxyl formations. Owing to the structural commonality of natural phenolic components, tannin, quinones, and flavonoids might play similar roles in tumor prevention and carcinogen metabolism regulation [84]. Quercetin, sideroxylin, formononetin, amentoflavone, and epigallocatechin gallate (EGCG) are included in flavonoids, which shared a basic structure with vitamin E. Sharing a three-ring structure, flavonoids can be classified into subgroups based on various substitutions in different positions. For instance, quercetin and sideroxylin are flavonols, formononetin belongs to isoflavonoids, amentoflavone is from polyflavonoids, and EGCG is included in proanthocyanidins. Each subgroup possesses a broad spectrum of in vitro activities including antioxidant, antitumor proliferative, and the characteristics to regulate enzymatic signaling pathways [85]. Moreover, the B ring of the flavonols owns a unique hydroxylation pattern, which reinforces the inhibition of mast cell secretion [86]. These findings implied that substitutions of the main structure should also be highlighted because they could offer distinct pharmacological functions and complementary roles. The substitutions in certain positions or the linkage among different parts could change the particular properties. The biomedical potential of natural compounds synchronously fluctuated with the reaction systems, because the human internal environment is extremely sophisticated [87]. Apart from that, the very existence of isomers should also be taken into consideration in natural phytochemicals [88,89]. 

Compared with multiple cellular studies demonstrating how natural plants compounds are applied in ovarian cancer, only a few mature animal models, such as MC38 murine models and xenograft models, have been explored in the present studies [90,91]. Therefore, the lack of large clinical approved trials is understandable. In these circumstances, deciphering the structure–activity relationship (SAR) of natural components may contribute to understanding the detailed pharmacology and pharmacochemistry, further identifying and mapping the cellular mechanisms. This could be the pointcuts of enhancing the bioavailability in human bodies, which can be applied to develop targeted therapeutic drugs and reduce side effects. Still, whether and how the antitumor potential is related to chemical structures remain to be explored, as well as how these structures may yet fulfill their unique attributes. In pharmacokinetics and pharmacological properties, a promising solution is to conjugate effective constituents together with carriers. These unique carriers are designed to target tumor tissue or immune cells specifically, such as silver nanoparticles, liposomes, polymers, micelles, polysaccharides, etc. [92]. Take polymer-based vesicular delivery platforms for example: they are macromolecular in order to recognize the physiology traits of tumors and raise up intratumoral bioavailability [93]. As for polysaccharides carriers, β-d-glucans have served as immune-adjuvants due to their modulatory properties in macrophages [94]. In clinical application, several natural herbs have been approved to treat ovarian cancer. The seed of brucea javanica oil has already been used in vivo, and its oil microemulsion and loaded liposomes forms are explored as improved formulations [95]. The encapsulated nanoparticles of curcumin enhanced the properties of drug release in the delivery system [96]. Nanocarrier quercetin validated its potential in various capsulate, namely, liposomes, antibody-conjugated micelles, and silver nanoparticles [97]. The milk exosomes augmented the concentration of berry Anthos in ovarian cancer patients [98]. There is a brand new co-delivery system that introduced both cisplatin and other drugs, which synergistically reversed cisplatin resistance and reduced side effects [99]. Taken together, these up-to-date strategies offered insight into the wild application of phytochemicals synergistic treatment in ovarian cancer.

## 6. Conclusions and Perspectives

Although first-line conventional chemotherapy is widely accepted to exert positive initial effects in many patients with ovarian cancer, drug resistance often hampers the following effective treatments. Natural botanical components have been exploited to apply for the prevention and adjuvant chemotherapy of ovarian cancer, which can specially improve the life quality of patients. As previous studies have demonstrated, numerous natural components are less toxic to healthy adjacent tissue and could mitigate tumor progression. The novel combination therapy strategy with natural drugs enhances the efficacy of chemotherapy and reduces toxic and side effects, thus prolonging the survival period of postoperative patients. The present review summarizes the antitumor mechanisms of plant-derived active ingredients in ovarian cancer, primarily their roles in the induction of apoptosis and autophagy, stimulation of ROS damage, inhibition of angiogenesis and metastasis, and reversing the MDR phenotype. In these circumstances, based on the natural new approaches in the form of nanostructures, chemical adjustment and synthetic production still merit further investigations [100]. Afterall, the underlying mechanisms of natural plant components for ovarian cancer are relatively complicated. Previous studies have paid attention to several classic signaling pathways, and only a few in vitro models have been explored. To comprehensively and systematically profile each accurate component, deeper research including pharmacology is warranted. However, there are still major drawbacks in current domestic and international research: (i) limited bioavailability and poor stability in vivo; (ii) the current mechanisms focused on certain parts of signaling pathways and lacked comprehensive molecular mechanisms; (iii) experimental animal model studies are not broadly applied; and (iv) lack of large, randomized, placebo-controlled clinical trials. Collectively, a breakthrough in these studies would significantly contribute to a better understanding of ovarian cancer pathologies, providing a theoretical basis for in-depth research and a reference for neoadjuvant chemotherapy in clinical application [101]. 

## Figures and Tables

**Table 1:** The antitumor effects of natural plants components in ovarian cancer.

Compound	Classification	Model	Effect	Mechanism	Reference
Tanshinones	Terpenoid/Abietane	A-549, TOV-21G	Cytotoxic action, ROS-JNK-CHOP, PI3K/AKT/mTOR signaling pathway	Reduce cell viability, inhibit colony formation capacity	[21,22,53]
Kadsuphilactone B	Terpenoid/Triterpene	A2780, Ishikawa cells	Cytotoxic action	Stimulate the activity of caspase-3/8/9 and MAPKs	[23]
Methyl lucidone (ML)	Oxo Steroid	OVCAR-8, SKOV-3	Cytotoxic action	Induce cellular apoptosis, stimulate the cleavage of caspase-3/9	[24]
Zeylenone (Zey)	Alicyclic	SKOV-3	JAK2/STAT3 signaling pathway	Reduce p-JAK and p-STAT expression level	[28]
Berbamine (BBM)	Alkaloid/Benzylisoquinoline	SKOV-3	Wnt/β-catenin signaling pathway	Increase activity of caspase-3/9, Bax and decrease Bcl-2 levels	[29]
Pinus massoniana bark proanthocyanidins (PMBP)	Phenol/Tannin	A-2780	NF-κB signaling pathway	Activate mitochondria-associated apoptosis	[30]
Sanguiin H-6 (SH-6)	Phenol/Tannin	A-2780	MAPK signaling pathway	Increase p15/BID level, activate MAPKs (p38)	[31]
Tetramethylpyrazine (TMP)	Alkaloid/Pyrazine	SKOV-3, OVCAR-3, A-2780 xenograft model	Suppress cell migration and invasion, reverse multiple-drug resistance (MDR)	Increase miR-211, diminish expression of proliferation and angiogenesis markers	[34,35,70]
Dihydroartemisinin (DHA)	Terpenoid/Artemisinin	HOSEPICs	Hedgehog signaling pathway	Inhibit cell malignant behaviors and augment apoptosis	[36]
Emodin	Quinone/Anthraquinones	A-2780, SKOV-3	ILK/GSK- 3β/Slug signaling pathway	Diminish the levels of ILK, p-GSK-3β, β-catenin, and Slug, repress EMT	[38]
Sulforaphane (SFN)	Ester/Isothiocyanate	Xenograft model, SKOV3-ip1, SKOV3TR-ip2	Promote DNA and ROS damage	Protein thiols oxidation, suppress cell viability, reduce hTERT and Bcl-2 levels	[40]
Quercetin	Flavonoid/Flavonol	SKOV-3, xenograft model	ROS-JNK-CHOP signaling pathway	Upregulate CHOP-induced DR5 expression following ROS damage	[42]
Formononetin (FMN)	Flavonoid/Isoflavone	ES2, OV-90	ERK1/2 signaling pathway	Upregulate caspase 3/9 and bax/bcl-2, interfere with sub G0/G1 phase arrest, increase P38 phosphorylation	[43]
Cucurbitacin-A	Terpenoid	SKOV-3	Trigger cell cycle arrest	DNA damage, ROS damage, MMP alterations, G2/M checkpoint	[46]
Asiatic acid	Terpenoid/Triterpene	SKOV-3, OVCAR-3	PI3K/Akt/mTOR signaling pathway	Downregulate phosphorylation levels of PI3K, AKT, and mTOR	[47]
Amentoflavone (AF)	Flavonoid	Xenograft model	ROS/AMPK/mTOR signaling pathway	Downregulate the expression of Skp2	[49]
Proanthocyanidins (BLPs)	Phenol/Tannin	A-2780, CP70, OVCAR-3 SP stem cell	Wnt/β-catenin, AKT/mTOR/p70S6K/4E-BP-1 signaling pathway	Reduce the expression of β-catenin, induce G1 cell cycle arrest, downregulate HIF-1α and VEGF	[50,58]
Scutellaria baicalensis (SB) and Fritillaria cirrhosa (FC)	Polysaccharides, Phenols, etc.	OVCA-420, OVCA-429	NF-κB signaling pathway	Activation of caspase-3 along with downregulation of cyclins D1 and D3	[51]
Grifolin	Terpenoid	A-2780, SKOV-3	AKT/mTOR/S6K signaling pathway	Upregulate autophagy markers LC3B, Atg7, Beclin-1, downregulate P62	[54]
Genistein	Terpenoid/sesquiterpenoid	OVCAR-3, SKOV-3	Inhibit VEGF levels	Reduce GD3 levels and restore NKT-cell functions	[56,71]
Isoliquiritigenin (ISL)	Ketone	OVCAR5, ES-2	Suppress cell viability	Increase cleaved PARP, caspase-3 and Bax/Bcl-2 ratio; trigger G2/M cell cycle phase	[57]
Flavonoids	Flavonoids	OVCAR-3	Inhibit angiogenesis	Downregulate VEGF in dose-dependent manner	[59]
Baicalin and baicalein	Flavonoid	OVCAR-3, CP-70	Inhibit angiogenesis	Suppress cancer promoting genes including VEGF, HIF-1α, cMy-c, and NF-kB	[61]
Harmine (HM)	Alkaloid	SKOV-3	Inhibit cell proliferation	Inhibit both the basal and EGF-induced phosphorylation levels of ERK1/2 and CREB	[62]
Cranberry proanthocyanidin-1 (PAC-1)	Phenol/Tannin	Chemotherapy-resistant SKOV-3, human umbilical vein endothelial cells (HUVEC)	Increase ROS generation, induce apoptosis	Block G2/M phase cell cycle progression, interfere with the phosphorylation level of VEGF-stimulated receptor	[63]
Astragalus polysaccharide (APS)	Polysaccharide	OV-90, SKOV-3	Inhibit proliferation and promote apoptosis	Upregulate miR-27a and FBXW7 expression levels	[64]
Sideroxylin	Flavonoid	ES2, OV-90	ERK1/2 signaling pathway	DNA and ROS damage, depolarize mitochondrial membrane depolarization, increase lipid peroxidation levels	[67]
Epigallocatechin gallate (EGCG)	Flavonoid	SKOV3-ip1, SKOV3TR-ip2	Promote DNA damage response	Suppress cell viability in time- and dose-dependent manner, reduce the expression of hTERT and Bcl-2	[68]
Berberine	Alkaloid	A2780, HEY, HO8910	Promote DNA damage response	Trigger oxidative DNA damages	[69]
Carotenoids	Terpenoid/Tetraterpenoid	Epidemiological statistics	Inhibit VEGF levels	Stimulate the activities of lymphocytes, macrophages, and cytotoxic T-cells	[72]
Lycopene	Terpenoid	OV-MZ-6	Diminish tumor load	Decrease expression of MMP9, ILK and EMT biomarkers	[73]
Deoxyschizandrin	Phenol/Tannin	A-2780	Increase ROS production	Inhibit macrophages, M2 phenotype markers CD163 and CD209	[74]
Curcumin	Phenol	SKOV3-ip1, HeyA8	Reduce mean tumor growth	Decrease microvessel density, increase tumor cell apoptosis	[75]
Procyanidin (GSP)	Phenol/Tannin	A-2780, SKOV-3	NF-κB and MAPK/ERK pathway	Augment cytotoxicity of paclitaxel and adriamycin	[76]

## References

[1] Webb P.M., Jordan S.J. (2017). Epidemiology of epithelial ovarian cancer. Best Pract. Res. Clin. Obstet. Gynaecol..

[2] Weidle U.H., Birzele F., Kollmorgen G., Rueger R. (2016). Mechanisms and Targets Involved in Dissemination of Ovarian Cancer. Cancer Genom. Proteom..

[3] Katabuchi H., Okamura H. (2003). Cell biology of human ovarian surface epithelial cells and ovarian carcinogenesis. Med. Electron Microsc..

[4] Duska L.R., Kohn E.C. (2017). The new classifications of ovarian, fallopian tube, and primary peritoneal cancer and their clinical implications. Ann. Oncol..

[5] Grunewald T., Ledermann J.A. (2017). Targeted Therapies for Ovarian Cancer. Best Pract. Res. Clin. Obstet. Gynaecol..

[6] Yoshida K., Miki Y. (2004). Role of BRCA1 and BRCA2 as regulators of DNA repair, transcription, and cell cycle in response to DNA damage. Cancer Sci..

[7] Bocchicchio S., Tesone M., Irusta G. (2019). Convergence of Wnt and Notch signaling controls ovarian cancer cell survival. J. Cell Physiol..

[8] Ediriweera M.K., Tennekoon K.H., Samarakoon S.R. (2019). Role of the PI3K/AKT/mTOR signaling pathway in ovarian cancer: Biological and therapeutic significance. Semin. Cancer Biol..

[9] Liu Z., Zhu Y., Li F., Xie Y. (2020). GATA1-regulated JAG1 promotes ovarian cancer progression by activating Notch signal pathway. Protoplasma.

[10] Chen Y., Bieber M.M., Teng N.N.H. (2014). Hedgehog signaling regulates drug sensitivity by targeting ABC transporters ABCB1 and ABCG2 in epithelial ovarian cancer. Mol. Carcinog..

[11] Dancey J. (2013). Targeted therapies and clinical trials in ovarian cancer. Ann. Oncol..

[12] Piccart M.J., Lamb H., Vermorken J.B. (2001). Current and future potential roles of the platinum drugs in the treatment of ovarian cancer. Ann. Oncol..

[13] Lheureux S., Gourley C., Vergote I., Oza A.M. (2019). Epithelial ovarian cancer. Lancet.

[14] Ottevanger P.B. (2017). Ovarian cancer stem cells more questions than answers. Semin. Cancer Biol..

[15] Coleman R.L., Monk B.J., Sood A.K., Herzog T.J. (2013). Latest research and treatment of advanced-stage epithelial ovarian cancer. Nat. Rev. Clin. Oncol..

[16] Wu J., Li Q.Q., Zhou H., Lu Y., Li J.M., Ma Y., Wang L., Fu T., Gong X., Weintraub M. (2014). Selective tumor cell killing by triptolide in p53 wild-type and p53 mutant ovarian carcinomas. Med. Oncol..

[17] Song K., Lv T., Chen Y., Diao Y., Yao Q., Wang Y. (2018). Emodin inhibits TGF-β2 by activating the FOXD3/miR-199a axis in ovarian cancer cells In Vitro. Oncol. Rep..

[18] Chen W., Lu Y., Chen G., Huang S. (2013). Molecular evidence of cryptotanshinone for treatment and prevention of human cancer. Anticancer Agents Med. Chem..

[19] Cullen S.P., Martin S.J. (2015). Fas and TRAIL ‘death receptors’ as initiators of inflammation: Implications for cancer. Semin. Cell Dev. Biol..

[20] Yuan X., Gajan A., Chu Q., Xiong H., Wu K., Wu G.S. (2018). Developing TRAIL/TRAIL death receptor-based cancer therapies. Cancer Metastasis Rev..

[21] Chang C.-C., Lai J.-S., Tsai C.-S., Ma S.-W., Lin J.-Y., Huang L.-R., Lu C.-H., Liao E.-C., Ho T.-F. (2013). Proapoptotic and TRAIL-sensitizing constituents isolated from Salvia militiorrhiza (Danshen). J. Biosci. Bioeng..

[22] Chang C.-C., Kuan C.-P., Lin J.-Y., Lai J.-S., Ho T.-F. (2015). Tanshinone IIA Facilitates TRAIL Sensitization by up-regulating DR5 through the ROS-JNK-CHOP Signaling Axis in Human Ovarian Carcinoma Cell Lines. Chem. Res. Toxicol..

[23] Jeong M., Kim H.M., Kim H.J., Choi J.-H., Jang D.S. (2017). Kudsuphilactone B, a nortriterpenoid isolated from Schisandra chinensis fruit, induces caspase-dependent apoptosis in human ovarian cancer A2780 cells. Arch. Pharm. Res..

[24] Yoon J.-H., Shin J.-W., Pham T.-H., Choi Y.-J., Ryu H.-W., Oh S.-R., Oh J.-W., Yoon D.-Y. (2020). Methyl lucidone induces apoptosis and G/M phase arrest the PI3K/Akt/NF-κB pathway in ovarian cancer cells. Pharm. Biol..

[25] Cheng X., Ferrell J.E. (2018). Apoptosis propagates through the cytoplasm as trigger waves. Science.

[26] Zhang L., Jin J., Zhang L., Hu R., Gao L., Huo X., Liu D., Ma X., Wang C., Han J. (2015). Quantitative analysis of differential protein expression in cervical carcinoma cells after zeylenone treatment by stable isotope labeling with amino acids in cell culture. J. Proteom..

[27] Zhang L., Huo X., Liao Y., Yang F., Gao L., Cao L. (2017). Zeylenone, a naturally occurring cyclohexene oxide, inhibits proliferation and induces apoptosis in cervical carcinoma cells via PI3K/AKT/mTOR and MAPK/ERK pathways. Sci. Rep..

[28] Xu X., Shi J., Gao H., Li Q. (2018). Zeylenone inhibits proliferation and promotes apoptosis in ovarian carcinoma cells via Janus kinase 2/signal transducers and activators of transcription 3 pathways. J. Obstet. Gynaecol. Res..

[29] Zhang H., Jiao Y., Shi C., Song X., Chang Y., Ren Y., Shi X. (2018). Berbamine suppresses cell proliferation and promotes apoptosis in ovarian cancer partially via the inhibition of Wnt/β-catenin signaling. Acta Biochim. Biophys. Sin..

[30] Liu J., Bai J., Jiang G., Li X., Wang J., Wu D., Owusu L., Zhang E., Li W. (2015). Anti-Tumor Effect of Pinus massoniana Bark Proanthocyanidins on Ovarian Cancer through Induction of Cell Apoptosis and Inhibition of Cell Migration. PLoS ONE.

[31] Lee D., Ko H., Kim Y.-J., Kim S.-N., Choi K.-C., Yamabe N., Kim K.H., Kang K.S., Kim H.Y., Shibamoto T. (2016). Inhibition of A2780 Human Ovarian Carcinoma Cell Proliferation by a Rubus Component, Sanguiin H-6. J. Agric. Food Chem..

[32] Lamouille S., Xu J., Derynck R. (2014). Molecular mechanisms of epithelial-mesenchymal transition. Nat. Rev. Mol. Cell Biol..

[33] Avila-Carrasco L., Majano P., Sánchez-Toméro J.A., Selgas R., López-Cabrera M., Aguilera A., González Mateo G. (2019). Natural Plants Compounds as Modulators of Epithelial-to-Mesenchymal Transition. Front. Pharmacol..

[34] Zhang H., Ding S., Xia L. (2021). Ligustrazine inhibits the proliferation and migration of ovarian cancer cells via regulating miR-211. Biosci. Rep..

[35] Yin J., Yu C., Yang Z., He J.-L., Chen W.-J., Liu H.-Z., Li W.-M., Liu H.-T., Wang Y.-X. (2011). Tetramethylpyrazine inhibits migration of SKOV3 human ovarian carcinoma cells and decreases the expression of interleukin-8 via the ERK1/2, p38 and AP-1 signaling pathways. Oncol. Rep..

[36] Liu Y., Gao S., Zhu J., Zheng Y., Zhang H., Sun H. (2018). Dihydroartemisinin induces apoptosis and inhibits proliferation, migration, and invasion in epithelial ovarian cancer via inhibition of the hedgehog signaling pathway. Cancer Med..

[37] Dong X., Fu J., Yin X., Cao S., Li X., Lin L., Ni J. (2016). Emodin: A Review of its Pharmacology, Toxicity and Pharmacokinetics. Phytother. Res..

[38] Lu J., Xu Y., Wei X., Zhao Z., Xue J., Liu P. (2016). Emodin Inhibits the Epithelial to Mesenchymal Transition of Epithelial Ovarian Cancer Cells via ILK/GSK-3/Slug Signaling Pathway. Biomed. Res. Int..

[39] Jiao R., Liu Y., Gao H., Xiao J., So K.F. (2016). The Anti-Oxidant and Antitumor Properties of Plant Polysaccharides. Am. J. Chin. Med..

[40] Kim S.C., Choi B., Kwon Y. (2017). Thiol-reducing agents prevent sulforaphane-induced growth inhibition in ovarian cancer cells. Food Nutr. Res..

[41] Sunil C., Xu B. (2019). An insight into the health-promoting effects of taxifolin (dihydroquercetin). Phytochemistry.

[42] Yi L., Zongyuan Y., Cheng G., Lingyun Z., Guilian Y., Wei G. (2014). Quercetin enhances apoptotic effect of tumor necrosis factor-related apoptosis-inducing ligand (TRAIL) in ovarian cancer cells through reactive oxygen species (ROS) mediated CCAAT enhancer-binding protein homologous protein (CHOP)-death receptor 5 pathway. Cancer Sci..

[43] Park S., Bazer F.W., Lim W., Song G. (2018). The O-methylated isoflavone, formononetin, inhibits human ovarian cancer cell proliferation by sub G0/G1 cell phase arrest through PI3K/AKT and ERK1/2 inactivation. J. Cell Biochem..

[44] Evan G.I., Vousden K.H. (2001). Proliferation, cell cycle and apoptosis in cancer. Nature.

[45] Jia S., Shen M., Zhang F., Xie J. (2017). Recent Advances in Momordica charantia: Functional Components and Biological Activities. Int. J. Mol. Sci..

[46] Liu J., Liu X., Ma W., Kou W., Li C., Zhao J. (2018). Anticancer activity of cucurbitacin-A in ovarian cancer cell line SKOV3 involves cell cycle arrest, apoptosis and inhibition of mTOR/PI3K/Akt signaling pathway. J. BUON.

[47] Ren L., Cao Q.-X., Zhai F.-R., Yang S.-Q., Zhang H.-X. (2016). Asiatic acid exerts anticancer potential in human ovarian cancer cells via suppression of PI3K/Akt/mTOR signalling. Pharm. Biol..

[48] Yu S., Yan H., Zhang L., Shan M., Chen P., Ding A., Li S.F.Y. (2017). A Review on the Phytochemistry, Pharmacology, and Pharmacokinetics of Amentoflavone, a Naturally-Occurring Biflavonoid. Molecules.

[49] Liu H., Yue Q., He S. (2017). Amentoflavone suppresses tumor growth in ovarian cancer by modulating Skp2. Life Sci..

[50] Zhang Y., Chen S., Wei C., Rankin G.O., Ye X., Chen Y.C. (2018). Dietary compound proanthocyanidins from Chinese bayberry (Myrica rubra Sieb. et Zucc.) leaves attenuate chemotherapy-resistant ovarian cancer stem cell traits via targeting the Wnt/β-catenin signaling pathway and inducing G1 cell cycle arrest. Food Funct..

[51] Kavandi L., Lee L.R., Bokhari A.A., Pirog J.E., Jiang Y., Ahmad K.A., Syed V. (2015). The Chinese herbs Scutellaria baicalensis and Fritillaria cirrhosa target NFκB to inhibit proliferation of ovarian and endometrial cancer cells. Mol. Carcinog..

[52] Lin L., Baehrecke E.H. (2015). Autophagy, cell death, and cancer. Mol. Cell Oncol.

[53] Zhou J., Jiang Y.-Y., Chen H., Wu Y.-C., Zhang L. (2020). Tanshinone I attenuates the malignant biological properties of ovarian cancer by inducing apoptosis and autophagy via the inactivation of PI3K/AKT/mTOR pathway. Cell Prolif..

[54] Che X., Yan H., Sun H., Dongol S., Wang Y., Lv Q., Jiang J. (2016). Grifolin induces autophagic cell death by inhibiting the Akt/mTOR/S6K pathway in human ovarian cancer cells. Oncol. Rep..

[55] Spagnuolo C., Russo G.L., Orhan I.E., Habtemariam S., Daglia M., Sureda A., Nabavi S.F., Devi K.P., Loizzo M.R., Tundis R. (2015). Genistein and cancer: Current status, challenges, and future directions. Adv. Nutr..

[56] Gossner G., Choi M., Tan L., Fogoros S., Griffith K.A., Kuenker M., Liu J.R. (2007). Genistein-induced apoptosis and autophagocytosis in ovarian cancer cells. Gynecol. Oncol..

[57] Chen H.-Y., Huang T.-C., Shieh T.-M., Wu C.-H., Lin L.-C., Hsia S.-M. (2017). Isoliquiritigenin Induces Autophagy and Inhibits Ovarian Cancer Cell Growth. Int. J. Mol. Sci..

[58] Zhang Y., Chen S., Wei C., Rankin G.O., Rojanasakul Y., Ren N., Ye X., Chen Y.C. (2018). Dietary Compound Proanthocyanidins from Chinese bayberry (Sieb. et Zucc.) leaves inhibit angiogenesis and regulate cell cycle of cisplatin-resistant ovarian cancer cells via targeting Akt pathway. J. Funct. Foods.

[59] Luo H., Jiang B.-H., King S.M., Chen Y.C. (2008). Inhibition of cell growth and VEGF expression in ovarian cancer cells by flavonoids. Nutr. Cancer.

[60] Dinda B., Dinda S., DasSharma S., Banik R., Chakraborty A., Dinda M. (2017). Therapeutic potentials of baicalin and its aglycone, baicalein against inflammatory disorders. Eur. J. Med. Chem..

[61] Chen J., Li Z., Chen A.Y., Ye X., Luo H., Rankin G.O., Chen Y.C. (2013). Inhibitory effect of baicalin and baicalein on ovarian cancer cells. Int. J. Mol. Sci..

[62] Gao J., Zhu H., Wan H., Zou X., Ma X., Gao G. (2017). Harmine suppresses the proliferation and migration of human ovarian cancer cells through inhibiting ERK/CREB pathway. Oncol. Rep..

[63] Kim K.K., Singh A.P., Singh R.K., Demartino A., Brard L., Vorsa N., Lange T.S., Moore R.G. (2012). Anti-angiogenic activity of cranberry proanthocyanidins and cytotoxic properties in ovarian cancer cells. Int. J. Oncol..

[64] Guo Y., Zhang Z., Wang Z., Liu G., Liu Y., Wang H. (2020). Astragalus polysaccharides inhibit ovarian cancer cell growth via microRNA-27a/FBXW7 signaling pathway. Biosci. Rep..

[65] Zhou J., Jiang Y.-Y., Wang X.-X., Wang H.-P., Chen H., Wu Y.-C., Wang L., Pu X., Yue G.-Z., Zhang L. (2020). Tanshinone IIA suppresses ovarian cancer growth through inhibiting malignant properties and angiogenesis. Ann. Transl. Med..

[66] Ai Z., Lu Y., Qiu S., Fan Z. (2016). Overcoming cisplatin resistance of ovarian cancer cells by targeting HIF-1-regulated cancer metabolism. Cancer Lett..

[67] Park S., Lim W., Jeong W., Bazer F.W., Lee D., Song G. (2018). Sideroxylin (Callistemon lanceolatus) suppressed cell proliferation and increased apoptosis in ovarian cancer cells accompanied by mitochondrial dysfunction, the generation of reactive oxygen species, and an increase of lipid peroxidation. J. Cell Physiol..

[68] Chen H., Landen C.N., Li Y., Alvarez R.D., Tollefsbol T.O. (2013). Epigallocatechin gallate and sulforaphane combination treatment induce apoptosis in paclitaxel-resistant ovarian cancer cells through hTERT and Bcl-2 down-regulation. Exp. Cell Res..

[69] Hou D., Xu G., Zhang C., Li B., Qin J., Hao X., Liu Q., Zhang X., Liu J., Wei J. (2017). Berberine induces oxidative DNA damage and impairs homologous recombination repair in ovarian cancer cells to confer increased sensitivity to PARP inhibition. Cell Death Dis..

[70] Tiper I.V., Temkin S.M., Spiegel S., Goldblum S.E., Giuntoli R.L., Oelke M., Schneck J.P., Webb T.J. (2016). VEGF Potentiates GD3-Mediated Immunosuppression by Human Ovarian Cancer Cells. Clin. Cancer Res..

[71] Milani A., Basirnejad M., Shahbazi S., Bolhassani A. (2017). Carotenoids: Biochemistry, pharmacology and treatment. Br. J. Pharmacol..

[72] Holzapfel N.P., Shokoohmand A., Wagner F., Landgraf M., Champ S., Holzapfel B.M., Clements J.A., Hutmacher D.W., Loessner D. (2017). Lycopene reduces ovarian tumor growth and intraperitoneal metastatic load. Am. J. Cancer Res..

[73] Lee K., Ahn J.-H., Lee K.-T., Jang D.S., Choi J.-H. (2018). Deoxyschizandrin, Isolated from Schisandra Berries, Induces Cell Cycle Arrest in Ovarian Cancer Cells and Inhibits the Protumoural Activation of Tumour-Associated Macrophages. Nutrients.

[74] Lin Y.G., Kunnumakkara A.B., Nair A., Merritt W.M., Han L.Y., Armaiz-Pena G.N., Kamat A.A., Spannuth W.A., Gershenson D.M., Lutgendorf S.K. (2007). Curcumin inhibits tumor growth and angiogenesis in ovarian carcinoma by targeting the nuclear factor-kappaB pathway. Clin. Cancer Res..

[75] Zhao B.-x., Sun Y.-b., Wang S.-q., Duan L., Huo Q.-l., Ren F., Li G.-f. (2013). Grape seed procyanidin reversal of p-glycoprotein associated multi-drug resistance via down-regulation of NF-κB and MAPK/ERK mediated YB-1 activity in A2780/T cells. PLoS ONE.

[76] Vinay D.S., Ryan E.P., Pawelec G., Talib W.H., Stagg J., Elkord E., Lichtor T., Decker W.K., Whelan R.L., Kumara H. (2015). Immune evasion in cancer: Mechanistic basis and therapeutic strategies. Semin. Cancer Biol..

[77] Costa-Rodrigues J., Pinho O., Monteiro P.R.R. (2018). Can lycopene be considered an effective protection against cardiovascular disease?. Food Chem..

[78] Liu M., Zhao S., Wang Z., Wang Y., Liu T., Li S., Wang C., Wang H., Tu P. (2014). Identification of metabolites of deoxyschizandrin in rats by UPLC-Q-TOF-MS/MS based on multiple mass defect filter data acquisition and multiple data processing techniques. J. Chromatogr. B.

[79] Vaidyanathan A., Sawers L., Gannon A.-L., Chakravarty P., Scott A.L., Bray S.E., Ferguson M.J., Smith G. (2016). ABCB1 (MDR1) induction defines a common resistance mechanism in paclitaxel- and olaparib-resistant ovarian cancer cells. Br. J. Cancer.

[80] Anand P., Sundaram C., Jhurani S., Kunnumakkara A.B., Aggarwal B.B. (2008). Curcumin and cancer: An “old-age” disease with an “age-old” solution. Cancer Lett..

[81] Zou L., Liu X., Li J., Li W., Zhang L., Li J., Zhang J. (2019). Tetramethylpyrazine Enhances the Antitumor Effect of Paclitaxel by Inhibiting Angiogenesis and Inducing Apoptosis. Front. Pharmacol..

[82] Yang F., Yu X.H., Qiao F., Cheng L.H., Chen G., Long X., Wang X.R., Li X.L., Liang R.C., Chen Y.Z. (2014). Formulation and characterization of Brucea javanica oil microemulsion for improving safety. Drug Dev. Ind. Pharm..

[83] Russo E.B. (2011). Taming THC: Potential cannabis synergy and phytocannabinoid-terpenoid entourage effects. Br. J. Pharmacol..

[84] Huang W.-Y., Cai Y.-Z., Zhang Y. (2010). Natural phenolic compounds from medicinal herbs and dietary plants: Potential use for cancer prevention. Nutr. Cancer.

[85] Kozłowska A., Szostak-Wegierek D. (2014). Flavonoids—Food sources and health benefits. Rocz Panstw Zakl Hig.

[86] Middleton E., Kandaswami C., Theoharides T.C. (2000). The effects of plant flavonoids on mammalian cells: Implications for inflammation, heart disease, and cancer. Pharmacol. Rev..

[87] Feng J., Zhang X.-L., Li Y.-Y., Cui Y.-Y., Chen Y.-H. (2016). Pinus massoniana Bark Extract: Structure-Activity Relationship and Biomedical Potentials. Am. J. Chin. Med..

[88] Cao Y., Himmeldirk K.B., Qian Y., Ren Y., Malki A., Chen X. (2014). Biological and biomedical functions of Penta-O-galloyl-D-glucose and its derivatives. J. Nat. Med..

[89] Wu S., Tian L. (2017). Diverse Phytochemicals and Bioactivities in the Ancient Fruit and Modern Functional Food Pomegranate (Punica granatum). Molecules.

[90] Hayakawa T., Yaguchi T., Kawakami Y. (2020). Enhanced anti-tumor effects of the PD-1 blockade combined with a highly absorptive form of curcumin targeting STAT3. Cancer Sci..

[91] Liao L., Liu C., Xie X., Zhou J. (2020). Betulinic acid induces apoptosis and impairs migration and invasion in a mouse model of ovarian cancer. J. Food Biochem..

[92] Mitra A., Nan A., Line B.R., Ghandehari H. (2006). Nanocarriers for nuclear imaging and radiotherapy of cancer. Curr. Pharm Des..

[93] Dragojevic S., Ryu J.S., Raucher D. (2015). Polymer-Based Prodrugs: Improving Tumor Targeting and the Solubility of Small Molecule Drugs in Cancer Therapy. Molecules.

[94] Su Y., Chen L., Yang F., Cheung P.C.K. (2021). Beta-d-glucan-based drug delivery system and its potential application in targeting tumor associated macrophages. Carbohydr. Polym..

[95] Ye H., Liu X., Sun J., Zhu S., Zhu Y., Chang S. (2016). Enhanced therapeutic efficacy of LHRHa-targeted brucea javanica oil liposomes for ovarian cancer. BMC Cancer.

[96] Hardwick J., Taylor J., Mehta M., Satija S., Paudel K.R., Hansbro P.M., Chellappan D.K., Bebawy M., Dua K. (2021). Targeting Cancer using Curcumin Encapsulated Vesicular Drug Delivery Systems. Curr. Pharm. Des..

[97] Vinayak M., Maurya A.K. (2019). Quercetin Loaded Nanoparticles in Targeting Cancer: Recent Development. Anticancer Agents Med. Chem..

[98] Aqil F., Jeyabalan J., Agrawal A.K., Kyakulaga A.-H., Munagala R., Parker L., Gupta R.C. (2017). Exosomal delivery of berry anthocyanidins for the management of ovarian cancer. Food Funct..

[99] Zhang M., Hagan C.T., Min Y., Foley H., Tian X., Yang F., Mi Y., Au K.M., Medik Y., Roche K. (2018). Nanoparticle co-delivery of wortmannin and cisplatin synergistically enhances chemoradiotherapy and reverses platinum resistance in ovarian cancer models. Biomaterials.

[100] Budisan L., Gulei D., Zanoaga O.M., Irimie A.I., Sergiu C., Braicu C., Gherman C.D., Berindan-Neagoe I. (2017). Dietary Intervention by Phytochemicals and Their Role in Modulating Coding and Non-Coding Genes in Cancer. Int. J. Mol. Sci..

[101] Halim C.E., Xinjing S.L., Fan L., Bailey Vitarbo J., Arfuso F., Tan C.H., Narula A.S., Kumar A.P., Sethi G., Ahn K.S. (2019). Anti-cancer effects of oxymatrine are mediated through multiple molecular mechanism(s) in tumor models. Pharmacol. Res..

